# Trans-cortical vessels in the mouse temporal bulla bone are a means to recruit myeloid cells in chronic otitis media and limit peripheral leukogram changes

**DOI:** 10.3389/fgene.2022.985214

**Published:** 2022-09-28

**Authors:** Ali Azar, Mahmood F. Bhutta, Jorge Del-Pozo, Elspeth Milne, Michael Cheeseman

**Affiliations:** ^1^ Developmental Biology Division, Roslin Institute and The Royal (Dick) School of Veterinary Studies, University of Edinburgh, Edinburgh, Scotland, United Kingdom; ^2^ Brighton and Sussex Medical School, Brighton, United Kingdom; ^3^ Department of ENT, Royal Sussex County Hospital, Brighton, United Kingdom; ^4^ Veterinary Pathology, The Royal (Dick) School of Veterinary Studies, University of Edinburgh, Edinburgh, Scotland, United Kingdom; ^5^ Division of Pathology, Institute of Genetics and Molecular Medicine, University of Edinburgh, Edinburgh, Scotland, United Kingdom; ^6^ Centre for Comparative Pathology, Division of Pathology, Institute of Genetics and Molecular Medicine, University of Edinburgh, Edinburgh, Scotland, United Kingdom

**Keywords:** trans-cortical vessels, otitis media, temporal bone, leukogram, *Mecom* ^*Jbo/+*^, *Fbxo11* ^*Jf/+*^, *Eda* ^*Ta*^

## Abstract

Chronic otitis media, inflammation of the middle ear, is a sequel to acute otitis media in ∼8% of children. Chronic otitis media with effusion is the most common cause of childhood deafness and is characterised by effusion of white blood cells into the auditory bulla cavity. Skull flat bones have trans-cortical vessels which are responsible for the majority of blood flow in and out of the bone. In experimental models of stroke and aseptic meningitis there is preferential recruitment of myeloid cells (neutrophils and monocytes) from the marrow in skull flat bones. We report trans-cortical vessels in the mouse temporal bone connect to the bulla mucosal vasculature and potentially represent a means to recruit myeloid cells directly into the inflamed bulla. The mutant mouse strains *Junbo* (*Mecom* ^*Jbo/+*^) and *Jeff* (*Fbxo11* ^*Jf/+*^) develop chronic otitis spontaneously; *Mecom* ^*Jbo/+*^ mice have highly cellular neutrophil (90%) rich bulla exudates whereas *Fbxo11* ^*Jf/+*^ mice have low cellularity serous effusions (5% neutrophils) indicating differing demand for neutrophil recruitment. However we found peripheral leukograms of *Mecom* ^*Jbo/+*^ and *Fbxo11* ^*Jf/+*^ mice are similar to their respective wild-type littermate controls with healthy bullae and infer preferential mobilization of myeloid cells from temporal bulla bone marrow may mitigate the need for a systemic inflammatory reaction. The cytokines, chemokines and haematopoietic factors found in the inflamed bulla represent candidate signalling molecules for myeloid cell mobilization from temporal bone marrow. The density of white blood cells in the bulla cavity is positively correlated with extent of mucosal thickening in *Mecom* ^*Jbo/+*^, *Fbxo11* ^*Jf/+*^, and *Eda* ^*Ta*^ mice and is accompanied by changes in epithelial populations and bone remodelling. In *Mecom* ^*Jbo/+*^ mice there was a positive correlation between bulla cavity WBC numbers and total bacterial load. The degree of inflammation varies between contralateral bullae and between mutant mice of different ages suggesting inflammation may wax and wane and may be re-initiated by a new wave of bacterial infection. Clearance of white blood cells and inflammatory stimuli from the bulla cavity is impaired and this may create a pro-inflammatory feedback loop which further exacerbates otitis media and delays its resolution.

## Introduction

The middle ear bulla is bounded by the ear drum (tympanic membrane) and the bulla bone which is lined by a mucosa attached to the bone periosteum. The air-filled bulla cavity contains the ossicular chain and the auditory (Eustachian) tube connects the air space to the nasopharynx. Inflammation of the middle ear, otitis media (OM), is characterised by fluid and white blood cell (WBC) effusion into the bulla cavity, changes in the mucosal lining and fluid accumulation in the bulla cavity which causes conductive hearing loss. Chronic otitis media with effusion (COME) affects 5–6% of children in high income countries in the second year of life and is the leading cause of childhood deafness (Bhutta, 2014). Chronic suppurative otitis media characterised by tympanic membrane perforation and discharge (otorrhea) affects 65–300 million people worldwide (Bhutta et al., 2017).

White blood cells enter the middle ear early in acute otitis media (AOM), with an initial infiltration of neutrophils and subsequently macrophages (Broides et al., 2002; Leichtle et al., 2011). In chronic inflammation, proteomic analysis of COME fluids from children undergoing tympanostomy tube placement shows upregulation of neutrophil products and neutrophil extracellular traps, as well as epithelial products such as mucins (e.g., MUC5B) and innate immunity proteins (e.g., BPIFA1). Pathway analysis shows upregulation of epithelial junctional remodelling and integrin signalling in the mucoid form of COME, and granulocyte adhesion and diapedesis in the serous presentation of COME (Val et al., 2016, 2018). Transcriptomic analysis of WBCs in the mucoid and serous forms of COME effusion shows upregulation of hypoxia/VEGF signalling, and Toll receptor, complement and RANKL/RANK pathways. Neutrophils and macrophages predominate in COME effusions but the serous form has increased lymphocyte populations judged by cytology and transcriptional cell signatures compared with mucoid COME (Bhutta et al., 2020).

The mucosa also shows changes in otitis media, including hyperplasia of columnar ciliated cells and goblet cells, polyp formation, infiltration of submucosal connective tissue with lymphocytes and neutrophils and cystic dilation of submucosal glands (Sade 1966; Magliocca et al., 2018). Mucosa associated lymphoid tissue (MALT) including lymphoid follicles is increased in the bulla and auditory tube mucosa of children with OM (Matsune et al., 1996; Kamimura et al., 2001).

White blood cells are not normally resident in the healthy bulla cavity, and the origin of those that accumulate is important to understanding otitis media pathogenesis. The systemic inflammatory response in otitis media is variable. Alterations in the leukogram such as leukocytosis and neutrophilia reflect the severity and nature of inflammatory response but are a variable feature of human AOM. Infants <2 months-old hospitalised with AOM can have an inflammatory leukogram, but >70% had normal WBC counts (Berkun et al., 2008). About 30% of children with AOM with bacterial-culture positive bulla fluids initially have a neutrophilia followed by neutropenia 10–14 days later (Schwartz et al., 1986) suggesting rapid consumption which exceeds the bone marrow storage pool. Significantly higher neutrophil counts are found with pneumococcal rather than non-typeable *Haemophilus influenzae* (NTHi) associated AOM (Polachek et al., 2004) and high grade mastoiditis is associated with neutrophilia (Bilavsky et al., 2009). Immature granulocytes are a feature of the leukogram in AOM, as a well as in many other acute inflammatory conditions such as upper and lower respiratory tract infections and gastroenteritis (Roehrl et al., 2011). This “shift to the left” is consistent with depletion of the mature neutrophils in the bone marrow storage pool and release of earlier stages. In one AOM study over 50% had children co-existing infections, the most common of which was bronchiolitis (Berkun et al., 2008). The severity of middle ear and tonsil infections were compared for the acute phase protein C-reactive protein, total WBC counts and absolute neutrophil counts and, unlike tonsillar infections, these markers were not correlated with severity in AOM (Christensen et al., 2013). Peripheral blood mononuclear cells including dendritic cells are higher in otitis-prone infants (Surendran et al., 2016). In human chronic OM, preoperative neutrophil-to-lymphocyte ratios were the same for patients undergoing tympanoplasty alone (inactive chronic OM group), or tympanoplasty and mastoidectomy (active chronic OM group) (Tansuker et al., 2017) and children undergoing tympanostomy tube placement did not have abnormal leukograms (Somuk et al., 2014).

Trans-cortical vessels (TCVs) comprising venules and arterioles are responsible for the majority of blood flow in and out of the bone in humans and mice. Recent work shows that in the mouse, myeloid cells (neutrophils and monocytes) are preferentially mobilized from the marrow in skull flat bones into experimental lesions mimicking stroke and aseptic meningitis (Herisson et al., 2018) and from tibial bone marrow into an experimental lesion mimicking chronic arthritis (Grüneboom et al., 2019).

The mouse temporal bone has a marrow cavity (Carretero et al., 2017) but the occurrence of TCVs has not been investigated. This vascular network could provide a direct route to recruit myeloid cells into the inflamed bulla and mitigate the need for a systemic immunological response. There are a number of well characterised mouse genetic strains that develop chronic OM (Bhutta et al., 2017). The use of chronic OM models may aid detecting haematological changes because recruitment of myeloid cells into the bulla is ongoing whereas in bacterial challenge models of AOM, inflammation of the bulla is self-limiting and any systemic haematological response may be short-lived. In this work we studied mutant mouse strains which develop otitis media spontaneously due to different underlying defects: *Junbo* (*Mecom* ^*Jbo/+*^) has dysregulated NF-kB-dependent inflammatory responses (Parkinson et al., 2006; Xu et al., 2012), *Jeff* (*Fbxo11* ^*Jf/+*^) has a bulla cavitation defect (Hardisty et al., 2003; Del-Pozo et al., 2019a) and *Tabby* (*Eda* ^*Ta*^) has deficit of nasopharyngeal submucosal glands (Azar et al., 2016; Del-Pozo et al., 2019b). We report that the mouse tympanic temporal bone has trans-cortical vessels which connect to the bulla mucosa and have characterised WBC populations in the bulla cavity and peripheral blood leukograms in *Mecom* ^*Jbo/+*^ and *Fbxo11* ^*Jf/+*^ mice. We found minimal changes in their peripheral blood leukograms which is consistent with preferential mobilization of myeloid cells from temporal bone marrow.

## Methods and materials

The animal experiments were reviewed and agreed by the Roslin Institute Animal Welfare and Ethical Review Body and were performed under the authority of an appropriate UK Home Office Licence; all experiments were performed in accordance with relevant guidelines and regulations. Heterozygous *Fbxo11* ^*Jf/+*^ mice (MGI, 1862017; European Mouse Mutant Archive, EM:00375) and their *Fbxo11* ^+/+^ wild-type littermates were generated by inter-crossing F1 *Fbxo11* ^*Jf*/+^ C57BL/6J C3H/HeH males with C57BL/6J (Charles River) females. Heterozygous *Mecom* ^*Jbo/+*^ mice (MGI, 2158381; EMMA EM:00091) and their wild-type littermate controls, *Mecom* ^*+/+*^, are congenic on a C3H/HeH genetic background. These strains were generated at the Mary Lyon Centre (MRC, Harwell) and imported into the Roslin Biological Resource Facility to establish breeding colonies. Tabby mice (*Eda* ^*Ta/Ta*^ females and *Eda* ^*Ta/Y*^ hemizygous males; collectively termed *Eda* ^*Ta*^) were maintained as a homozygous line. FVB mice are the background inbred genetic line for the *Eda* ^*Ta*^ strain and FVB/NCrl (Charles River) mice were bred to provide control tissues. Mouse husbandry, genotyping, health surveillance and health status is reported elsewhere (Azar et al., 2016; Del-Pozo et al., 2019a; Del-Pozo et al., 2019b) and is reported in accordance with ARRIVE guidelines.

### Sample collection

Mice were euthanized using a rising concentration of CO_2_. FACS analysis was performed on blood collected by cardiac puncture, spleen tissue and on a PBS wash for peritoneal cells (see below). There are well documented genetic strain differences for total blood WBC counts and WBC differentials (Peters et al., 2002) which require consideration because *Mecom* mice have a C3H/HeH genetic background and *Fbxo11* mice have a mixed C57BL/6J C3H/HeH background. In addition there are within strain sex differences (Peters et al., 2002). We analysed blood and spleen WBC samples from P98-P147 *Mecom* ^*Jbo/+*^ (5 females, 3 males); *Mecom* ^*+/+*^ (4 females, 3 males); P105-P119 *Fbxo11* ^*Jf/+*^ (3 females, 3 males); *Fbxo11* ^*+/+*^ (3 females, 3 males). We standardized collection by making it at same time each morning.

In these mouse mutants OM is generally bilateral but can be unilateral. Wherever possible a single bulla effusion sample was taken from each ear and represents a biological replicate. To collect bulla fluids, the head was skinned and the tympanic membrane (TM) visualised under ×10 binocular magnification and LED illumination. A small proportion of *Mecom* ^*Jbo/+*^ mice have a thin crust-like layer overlying the TM that has to be removed with forceps before making a small hole in the TM with a clean pair of fine forceps. Each bulla fluid was collected into 200 µl of sterile ice-cold PBS by injecting a 2 µl aliquot through this TM perforation and gently flushing in and out 3 times. The pipette tip was positioned just inside the bulla cavity to avoid damaging the mucosa. This rinse was repeated five times, each time adding the cell suspension back to the 200 µl sample. The PBS sample was gently mixed using a micropipette and 175 µl aliquots were used for FACS. Bulla fluid samples used for FACS were collected from P98-P147 *Mecom* ^*Jbo/+*^ (*n* = 12 bulla samples from 4 female, 3 male) and P105-P119 *Fbxo11* ^*Jf/+*^ (*n* = 14 bulla samples from 3 female and 5 male) mice.

Bulla fluid samples (see above) for WBC haemocytometer counts and bacteriology were collected from a separate cohort of P95-P123 *Mecom* ^*Jbo/+*^ (22 bulla samples from *n* = 8 females, *n* = 3 males) and P95-P102 *Fbxo11* ^*Jf/+*^ mice (36 bulla samples from *n* = 8 females, *n* = 10 males). Ten µl of the sample was used for a haemocytometer count and 50 µl aliquots of the *Mecom* ^*Jbo/+*^ samples were cultured for aerobic bacteria as previously described (Azar et al., 2016).

### Histology and morphometric analysis

For wax histology, a separate collection of skinned heads (see figure legends for ages and numbers) were immersion fixed in neutral buffered formalin and decalcified for 2–4 days in 14% EDTA in universal tubes on a roller mixer. The trimmed skulls were processed and embedded in wax with the ventral surface (hard palate side) down and sectioned in dorsal plane to reveal the bullae. Specimens were then re-blocked in groups of 3 or 4 for further sectioning. Four μm sections were cut and stained with either Haematoxylin and Eosin (H–E) or Alcian Blue-Periodic Acid Schiff (AB-PAS). Immunohistochemistry was performed for laminin (Del-Pozo et al., 2019a).

Bright field images were acquired on an Olympus BX41 microscope equipped with a DP72 camera and Cell D software. Slide scans were made using a Hamamatsu NanoZoomer were analysed with NanoZoomer software and with Qu-Path software (Bankhead et al., 2017).

For morphometric analysis we selected a standardized dorsal plane section of tympanic part of the temporal bone that was rostral the cochlea promontory (petrous part of the temporal bone) and ventral to the auditory tube ostia. A 1–3 mm length of mucosa over the tympanic temporal bone from its suture with petrous temporal bone was assessed for mucosal thickness by dividing area of tissue over a defined length of underlying bulla bone. The mucosal area includes epithelium cell bodies (excluding cilia), submucosal connective tissue, blood and lymphatic vessels and periosteum. To examine the relationship between bulla mucosal thickness and the number of bulla cavity WBCs we made a single H–E stained section for each bulla measuring mucosal thickness (as above) and enumerated bulla cavity WBCs as the number of cells (objects) using the cell analysis mode of Qu-Path software (Bankhead et al., 2017) which distinguishes nucleated cells from non-nucleated RBCs. In our experience this analysis makes a robust assessment of nucleated cells with karyorrhectic nuclei, and assigns a cluster karyorrhectic fragments to single a nucleated cell. The sample numbers and mouse genotypes for these analyses are given in the figure legend.

Epithelial cell types (ciliated, non-ciliated and goblet cells) were counted in dorsal pole (TM to stapedial artery avoiding the oval window), promontory (stapedial artery to the suture between petrous and temporal bone) and mesotympanum (aforementioned suture to TM). The level selected was ventral to the auditory tube ostium which has anatomically normal goblet cells. Cells were counted in a pair of inflamed P57 *Eda* ^*Ta*^ bullae and a pair of P79 FVB bullae as a healthy normal control.

Tympanic temporal bone morphometric analysis was performed on P79-P90 *Eda* ^*Ta*^ and P81-P84 FVB specimens. Bone thickness measurements were made in a ∼0.5 mm region located in mesotympanum at a mid-point between promontory and auditory tube ostia, level with the branch of the auricular artery. The channel prevalence (presence versus absence in a tissue section) and channel widths were measured on the mucosal bone cortex and (contralateral) medial surface. Some temporal bones were excluded for certain measurements because the section level was incorrect or the cortex was artefactually damaged. Temporal bone sample size for each assay is given in the Figures.

### FACS analysis of WBCs bulla fluid wash, spleen and peripheral blood

Single cell suspension of PBS bulla fluid wash, spleen and peripheral blood were stained with a cocktail of antibodies containing CD45, F4/80, Ly6c and Ly6g, to characterise the main subsets of myeloid lineages: neutrophils, monocytes and macrophages. For lymphoid T and B cell population analysis a cocktail of antibodies containing CD45, B220, and CD5 markers was used and the reagents are detailed elsewhere (Azar et al., 2016).

### Liposome Dil and clodronate treatments

Liposome reagents from Liposoma, the Netherlands (clodronateliposomes.com) were administered by intraperitoneal (i.p.) injection. In a pilot experiment to investigate liposome uptake in macrophages and monocyte populations in target tissues we used fluorescent Dil liposomes. These were administered by a single 100 µl i.p. injection in >P56 *Mecom* ^*Jbo/+*^ mice and analysed in peripheral blood and single cell PBS suspensions of peritoneal wash, spleen and bulla cavity washes made from individual mice euthanized at either 4 h (*n* = 4), 24 h (*n* = 4), or 48 h (*n* = 3) post-injection, and in non-injected controls (*n* = 3). To investigate systemic depletion of macrophages and monocytes in bulla populations, clodronate liposomes (or control PBS liposomes) were administered to P112-P126 *Mecom* ^*Jbo/+*^ mice by two 100 µl i.p. injections at three or 4-days intervals; *n* = 14 mice were in the clodronate treatment group and *n* = 10 mice were in the PBS control group. Single cell suspensions were made from peritoneal and bulla cavity washes. FACS analysis of CD45^+^ live cells in 175 ul aliquots were gated for macrophages and monocytes versus neutrophils. The Dil fluorescent signal was measured according to the manufacturer’s instruction. The macrophage and monocyte populations were expressed as a percentage of CD45^+^ cells.

### Statistical analysis and graphical representation

The statistical tests were chosen after D'Agostino and Pearson or Shapiro-Wilk normality tests, and are given in results and/or figure legends. Graphs represent data as either points, histogram bars with the appropriate parametric or non-parametric summary statistics, or by Tukey method box-and-whisker plots; the box represents the 25% percentile, the median and the 75% percentile, the whiskers the minimum and the maximum and the points outliers.

Histological data on WBC numbers in the bulla cavity and mucosal thickness were each analysed in *Mecom* ^*Jbo/+*^, *Fbxo11* ^*Jf/+*^, and *Eda* ^*Ta*^ and their respective wild-type controls. An arbitrary value of one was given to zero WBC counts for graphical representation on a logarithmic scale. Spearman rank correlation tests were performed on paired WBC and mucosal thickness data, and on WBC and bacterial count data from individual bulla samples. Fisher’s Exact or Chi-square contingency tests were performed on frequency data. Frequency data for epithelial cell types in *Eda* ^*Ta*^ and FVB bullae were graphed as proportions and analysed by Chi-square tests; the raw cell counts and Chi-square tests are presented in Supplementary Table S1.

FACS data for *Fbxo11* ^*Jf/+*^ and *Mecom* ^*Jbo/+*^ bulla WBCs were analysed using Kruskal–Wallis tests followed by Dunn’s multiple comparison tests, for following cell classes: myeloid cells (neutrophils, MΦ, total monocytes); Ly6c^High^ and Ly6c^+^ monocyte subsets; T and total B cells.

Temporal bone channel width data for FVB and *Eda* ^*Ta*^ was analysed in a 2-way ANOVA with Sidak’s multiple comparisons tests for genotype and cortex surface.

FACS data for blood and spleen cells were analysed in a series of 2-way ANOVAs with Tukey’s or Sidak’s multiple comparisons tests for genotype (*Fbxo11* ^*Jf/+*^, *Fbxo11* ^+/+^, *Mecom* ^*Jbo/+*^, and *Mecom* ^+*/+*^) cell classes: myeloid cells (neutrophils, MΦ, total monocytes); Ly6c^High^ and Ly6c^+^ monocyte subsets; total T and total B cells; and B1 and IgD^+^ B cell subsets. Two-tailed tests were used throughout and test values *p* < 0.05 were considered to be statistically significant. Graphs and statistics were generated using Prism Graph Pad.

## Results

### Tympanic temporal bone marrow and trans-cortical vessel connections to bulla mucosa

**FIGURE 1:**
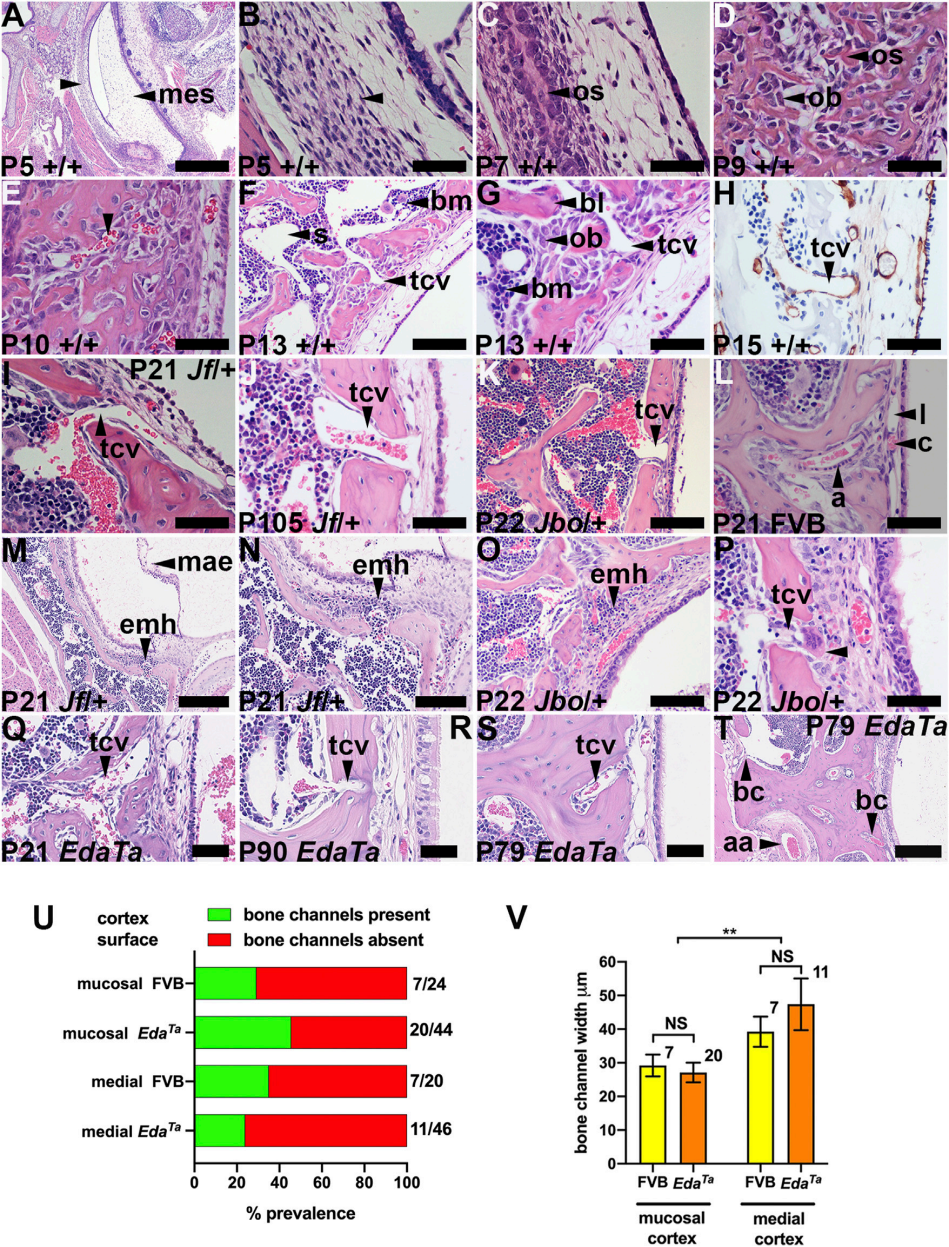
Temporal bone trans-cortical vessels connect bone marrow to bulla mucosa. Dorsal plane sections rostral uppermost. **(A,B) (F,G)**, and **(M,N)** are low and high magnification images of the same section. **(A–H)** Intramembranous ossification of tympanic temporal bone in wild-type mice (mixed C57BL/6J and C3H/HeH genetic background). **(A,B)** P5 bone primordium consists of mesenchymal osteoprogenitor cells. Osteoblast and osteoid at **(C)** P7 and **(D)** P9, **(E)** P10 bone marrow vascular channels (arrowhead), **(F,G)** P13 lamellar bone formation by osteoblasts, bone marrow and sinuses, and TCV extending through a bone channel. **(H)** TCV basal lamina stains for laminin. **(I–L)** TCVs connecting marrow with mucosa. **(L)** P21 FVB mucosa has capillary and lymphatic vessels and an arteriole bordered by pericytes. **(M-P)** Extra medullary haematopoiesis in mucosa. **(P)** A megakaryocyte (arrowhead) in a periosteal location. TCV in **(Q)** P21 *Eda* ^*Ta*^ mice extends through the full thickness of the cortex. **(R,S)** P90 and P79 *Eda* ^*Ta*^ TCVs in a deep pocket in the bone cortex. **(T)** A wide bone channel in the medial bone cortex (upward arrowhead) and a narrower one in the mucosal bone cortex (downward arrowhead); branch of the auricular artery on the medial face of the mesotympanum bone cortex. **(U)** The prevalence of bone channels in does not differ significantly between mucosal and medial surfaces of temporal bone or between FVB and *Eda* ^*Ta*^ mice (*p* = 0.176; 2 x 4 Chi-square test). The fractions beside each histogram bar are the number of sections in which channels are observed/number of sections examined. **(V)** Bone channel width is comparable FVB and *Eda* ^*Ta*^ mice, but those in the medial cortex are larger than the mucosal cortex (*p* = 0.0072; Two-Way ANOVA). Histogram bars represent the mean and the error bars +/− s.e.m.; the number beside the histogram bar represents the number of channels measured. Scale Bars: **(A)** 500 μm; **(T)** 250 μm; **(M)** 200 μm; **(F,N,O,K)** 100 μm; **(B-E,G-J,L,P-S)** 50 µm.aa, auricular artery; a, arteriole; bc, bone channel; bl, bone lamella; bm, bone marrow; c, capillary; emh, extramedullary haematopoiesis; l, lymphatic vessel; mae, mesenchyme associated epithelium; mes, mesenchyme; ob, osteoblast; os, osteoid; s, bone marrow sinus; tcv, trans-cortical vessel.

The petrous part of the murine temporal bone contains the vestibulocochlear organ (Navarro et al., 2017) and develops from an embryonic cartilaginous primordium at embryonic (E) day E12.5-13 then undergoes endochondral ossification at post-natal day 5 (P5) (Graham et al., 2015). The tympanic membrane attaches to ectotympanic ring, and this part of the tympanic temporal bone is calcified at P0. The remainder of the tympanic temporal bone undergoes intramembranous ossification and this is complete by P9 (Richter et al., 2010). We found the tympanic temporal bone is a mesenchymal primordium at P5 (Figures 1A,B) which undergoes intramembranous ossification by P7 (Figures 1C,D) and develops blood filled vessels by P10 (Figure 1E). By P13 and P15 there is osteoid remodelling and mineralization forming spicules and trabeculae with bone marrow in the inter-trabecular spaces (Figures 1F,G). At P21 the periosteal osteoblasts form a lamellar bone cortex.

The tympanic temporal bone has a continuous marrow cavity dorsally, but marrow cavity is restricted to the bone extremities in the hypotympanum. The P21 mucosal cortical bone surface has a slender periosteum and cortical bone channels that contain trans-cortical vessels (TCVs) (Figures 1F–K,P,Q). TCVs extend from marrow sinuses and are thin walled venules with a laminin positive basement lamina (Figure 1H). There are also occasional arterioles which appear to emerge from the cortex (Figure 1L). Cortical bone channels are also found on the contralateral (medial) cortex. The cortical bone channels illustrated in Figures 1F–L,P,Q are in a favourable plane of section that shows they extend through the full-thickness of the cortex; others appear deep pocket containing a TCV (Figures 1R,S). Bone channels in the medial cortex are large (Figure 1T). The bone channel prevalence is not significantly different between mucosal and medial cortices, or comparing inflamed P79-P90 *Eda* ^*Ta*^ and healthy P81-P84 FVB tympanic temporal bones (Figure 1U). Bone channel size is comparable between genotypes, but those of the medial cortex are significantly larger (Figure 1V).

Mucosal vessels include arterioles, capillaries and lymphatics. In wild-type mice bulla cavitation occurs at ∼ P11/P12 but this process is defective in *Fbxo11* ^*Jf/+*^ mice (Del-Pozo et al., 2019a). In *Mecom* ^*Jbo/+*^, *Fbxo11* ^*Jf/+*^ and *Eda* ^*Ta*^ OM initiates after bone marrow development at ∼ P13 and, unlike wild-type mice, there are foci of extra medullary haematopoiesis in the deep mucosa at P21 (Figures 1M–P).

### WBC differential counts in bulla fluids, blood and spleen in *Mecom* and *Fbxo11* mice

**FIGURE 2:**
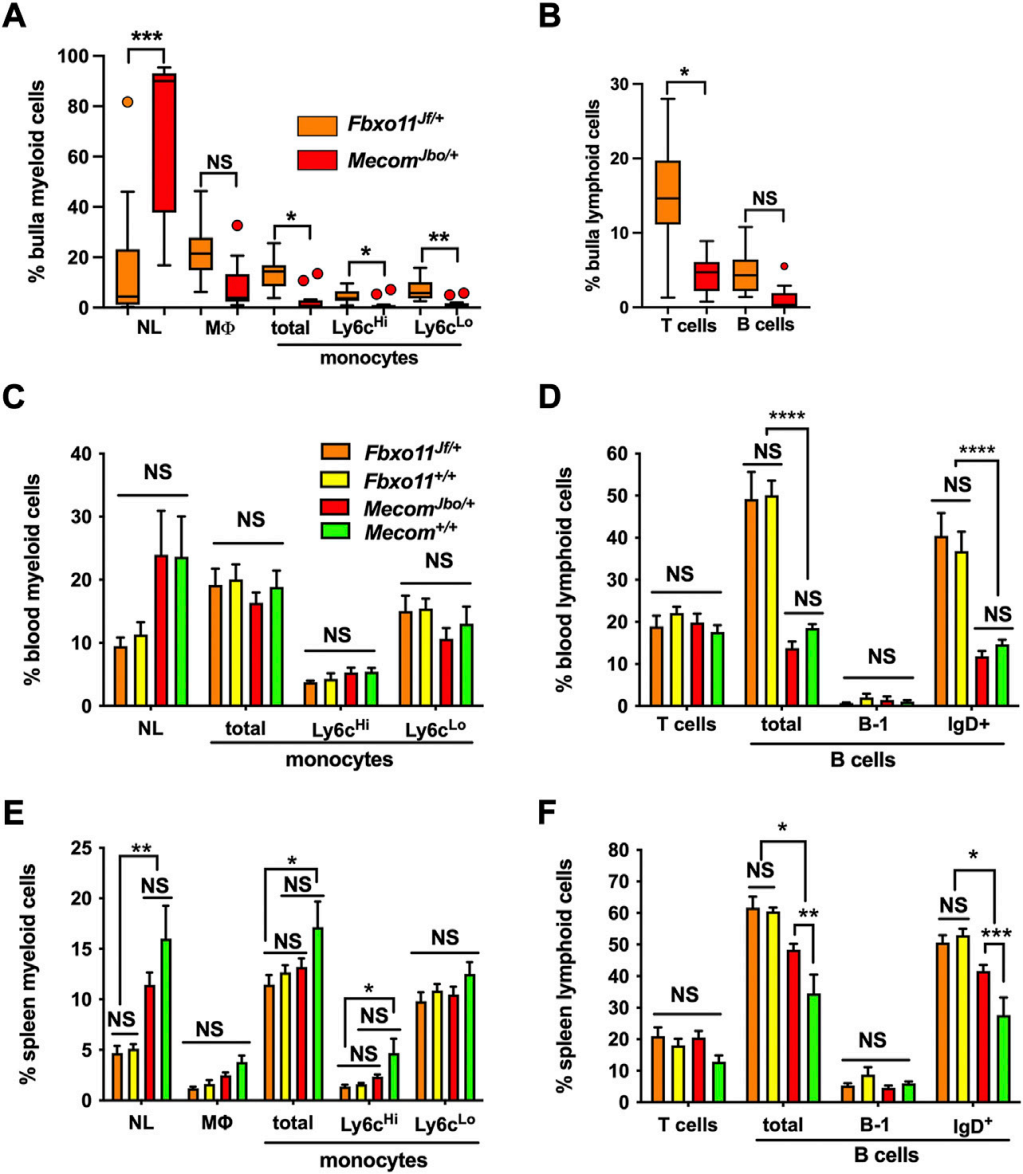
FACS analysis of bulla fluid, blood and spleen WBC. **(A,B)** Bulla fluids **(C,D)** blood and **(E,F)** spleen.**(A)** Bulla fluids in *Mecom* ^*Jbo/+*^ mice have a significantly higher percentage of neutrophils than *Fbxo11* ^*Jf/+*^ mice, whereas *Fbxo11* ^*Jf/+*^ mice have higher total monocytes, higher Ly6c^Hi^ and Ly6c^Lo^ monocyte subsets and **(B)** higher T cells than *Mecom* ^*Jbo/+*^ mice. Data in A and B are for *n* = 14 *Fbxo11* ^*Jf/+*^ and *n* = 12 *Mecom* ^*Jbo/+*^ bulla fluids and are represented by box-and-whisker plots, with outlier vales represented by points, and analysed in a series of Kruskal–Wallis tests followed by Dunn’s multiple comparison tests (see statistical analysis for details).**(C)** Blood myeloid and **(D)** lymphoid cells. Within mouse strains, myeloid and lymphoid cells are not significantly different in *Mecom* ^*Jbo/+*^ and *Mecom* ^*+/+*^ comparisons, and in *Fbxo11* ^*Jf/+*^ and *Fbxo11* ^+/+^ comparisons. There were genetic background strain differences between *Fbxo11* and *Mecom* leukograms; *Fbxo11* mice had higher total B lymphocytes and IgD^+^ B subset populations.**(E)** Spleen myeloid and **(F)** lymphoid cells. Within mouse strains, *Mecom* ^*Jbo/+*^ mice had higher total splenic B lymphocytes and IgD^+^ B subsets than *Mecom* ^*+/+*^ mice. All other myeloid and T cells comparisons in *Mecom* ^*Jbo/+*^ and *Mecom* ^*+/+*^, and in *Fbxo11* ^*Jf/+*^ and *Fbxo11* ^+/+^ were not significantly different. Genetic background strain differences include *Fbxo11* mice having higher total B lymphocytes and IgD^+^ B subset populations and *Mecom* mice have higher splenic neutrophils than *Fbxo11* mice and *Mecom* ^*+/+*^ mice have higher total monocytes and Ly6c^Hi^ monocyte subsets than *Fbxo11* ^*Jf/+*^ mice (Figure 2E). Data in panels **(C–F)** are for *n* = 6 *Fbxo11* ^*Jf/+*^, *n* = 6 *Fbxo11* ^*+/+*^, *n* = 8 *Mecom* ^*Jbo/+*^, and *n* = 7 *Mecom* ^*+/+*^ samples; the histogram bars represent mean and the error bar s.e.m. Blood and spleen WBC differentials were analysed in a series of two-way ANOVA’s with Tukey’s or Sidak’s multiple comparisons tests (see statistical analysis for details). Two-tailed tests: NS, not significant *p* > 0.05, *p < 0.05, **p < 0.01, ***p < 0.001, ****p < 0.0001.

To assess bulla WBC populations and the wider context of blood and spleen we performed FACS analysis in mutant and wild-type littermate controls. The gating strategy for bulla WBCs is shown in Supplementary Figures S1A–G. Bulla fluids in *Mecom* ^*Jbo/+*^ mice have significantly higher percentage of neutrophils than *Fbxo11* ^*Jf/+*^ mice (medians of ∼90% and ∼5% respectively), whereas *Fbxo11* ^*Jf/+*^ mice have higher total monocytes, higher Ly6c^Hi^ inflammatory monocyte subsets and higher T cells than *Mecom* ^*Jbo/+*^ mice (Figures 2A,B). However each cohort contained outliers such that some *Fbxo11* ^*Jf/+*^ bullae had high neutrophils. *Mecom* ^*Jbo/+*^ bulla fluids are suppurative exudates which have a higher proportion of neutrophils than macrophages and monocytes (*p* < 0.001, Kruskal-Wallis tests and Dunn’s multiple comparison tests), whereas the neutrophil, macrophage and monocyte populations are not significantly different in the serous bulla effusions of *Fbxo11* ^*Jf/+*^ bullae and could be classified as subacute inflammation.

Haemocytometer counts of bulla fluid WBCs were ∼10 times higher in *Mecom* ^*Jbo/+*^ (median 766,000, 25%–75% percentile 327,500–1,368,000, *n* = 22) than *Fbxo11* ^*Jf/+*^ mice (median 76,000, 25%–75% percentile 33,500–152,250, *n* = 36; Mann-Whitney test *p* < 0.0001). We cultured *Mecom* ^*Jbo/+*^ bulla fluids; 5 of 22 samples had no growth of aerobic bacteria. *Proteus mirabilis*, DNAse neg *Staphylococcus* sp., *E. coli*, *Gemella* spp. and *Enterococcus* sp. were isolated from the other cases either in pure or mixed culture. The median total bacterial load was 3.7 × 10^3^ CFU (range 5 × 10^1^ to 1.5 × 10^6^ CFU, *n* = 17). There was a significant positive correlation between WBC numbers and total bacterial load (Spearman r = 0.527 *p* = 0.0117 *n* = 22 XY pairs).

Within mouse strains, *Mecom* ^*Jbo/+*^ mice had higher total splenic B lymphocytes and IgD^+^ B subsets than *Mecom* ^*+/+*^ mice. All other WBC cell classes in blood (Figures 2C,D) and spleen (Figures 2E,F) in *Mecom* ^*Jbo/+*^ and *Mecom* ^*+/+*^, and in *Fbxo11* ^*Jf/+*^ and *Fbxo11* ^+/+^ comparisons were not significantly different suggesting that OM is not associated with substantial peripheral blood leukogram or spleen changes.

There are however genetic background strain differences between *Fbxo11* and *Mecom.* For example *Fbxo11* mice had higher total lymphocytes and IgD^+^ B subset populations in blood (Figure 2D) and spleen (Figure 2F) whereas *Mecom* mice have higher splenic neutrophils than *Fbxo11* mice and *Mecom* ^*+/+*^ mice have higher total monocytes and Ly6c^Hi^ monocyte subsets than *Fbxo11* ^*Jf/+*^ mice (Figure 2E).

### Systemic depletion of macrophage and monocytes does not deplete *Mecom* ^*Jbo/+*^ bulla cavity populations in the short term

Intrabullar injection of *Haemophilus influenzae* in mice that are depleted of macrophages by CCR2 deficiency and chlodronate liposomes results in prolonged neutrophilic infiltration in the middle ear mucosa and lumen and delayed clearance of infection (Hur et al., 2021). We were interested to explore the role of macrophage depletion in neutrophil clearance in the fluid filled bulla cavity. We first tested the uptake of Dil fluorescent labelled liposomes by target populations. In a 48 h time course, macrophage and monocyte populations in the peritoneum cavity, spleen and blood took up tracer Dil liposomes, but bulla populations did not (Supplementary Figures S2A–D). We next depleted systemic populations with two cycles of clodronate liposome treatment administered at 3–4 days intervals. This treatment reduced peritoneal populations from a median of 30% to 7% (Supplementary Figure S2E) but there was no detectable depletion of bulla cavity macrophage and monocyte populations or a change in neutrophil populations when expressed as percentage of CD45^+^ cells (Supplementary Figures S2F,G).

### Bulla mucosal thickness and effusion WBCs are positively correlated

**FIGURE 3:**
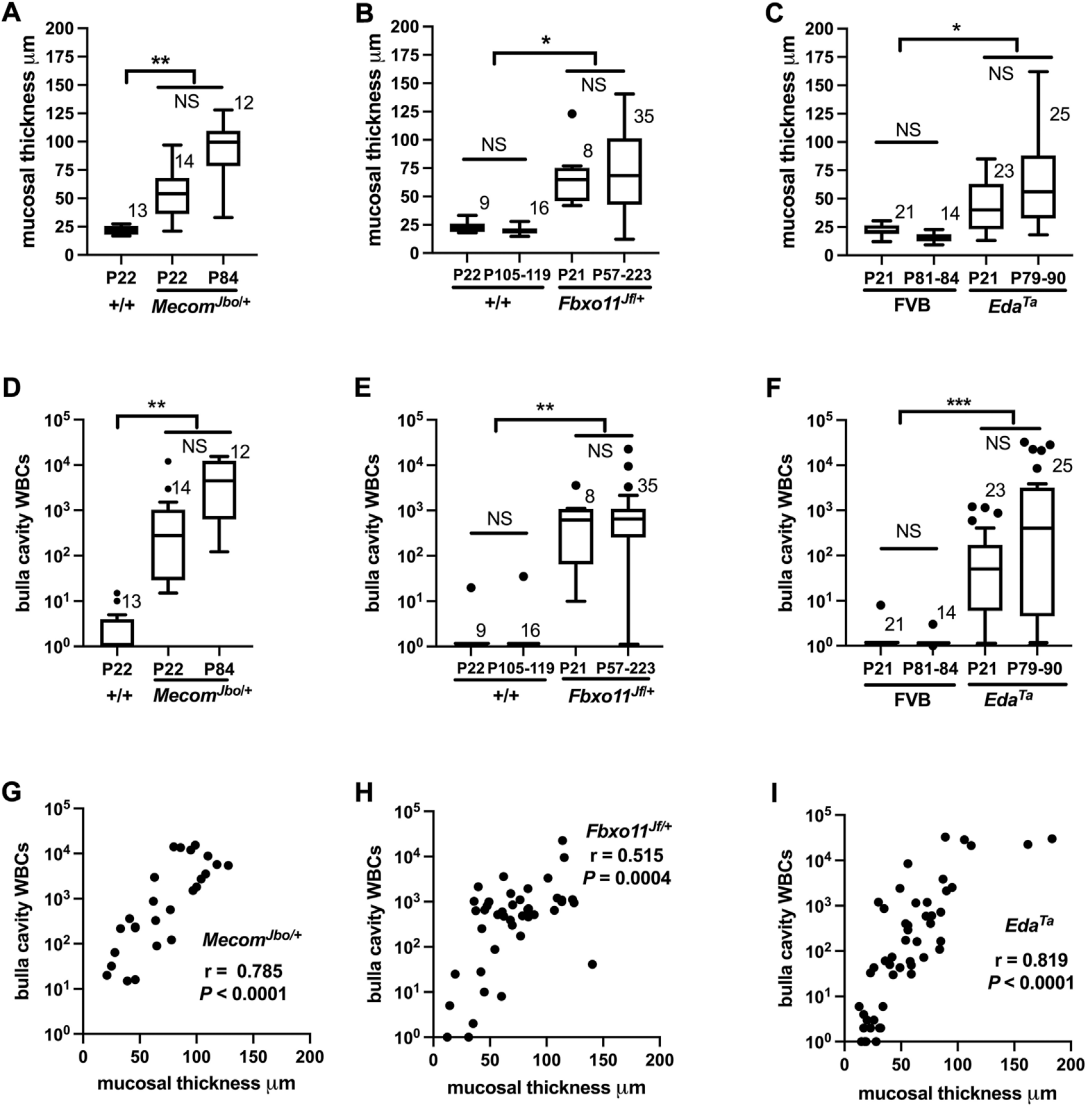
WBC numbers in the bulla cavity and mucosal thickness are positively correlated in *Mecom* ^*Jbo/+*^, *Fbxo11* ^*Jf/+*^, and *Eda* ^*Ta*^ mice. Morphometric analysis of a single **(H–E)** stained section made for each individual bulla. Mucosal thickness **(A–C)**, and bulla cavity WBCs **(D–F)** in *Mecom* ^*Jbo/+*^, *Fbxo11* ^*Jf/+*^, and *Eda* ^*Ta*^ mice are increased compared with wild-type controls. However the trends for increased mucosal thickness or bulla WBCs between the initiation of OM (P21, weaning age) and in older mice with a chronic lesion (>P54) do not achieve statistical significance. Data in graphs A–F are represented by box-and-whisker plots. The number to the right of the box indicates the number of bulla samples in each group. Data were analysed using Kruskal–Wallis tests followed by Dunn’s multiple comparison tests. Two-tailed tests: NS not significant *p* > 0.05, *p < 0.05, **p < 0.01, ***p < 0.001. **(G–I)** In individual bullae, the bulla cavity WBC numbers and mucosal thickness are positively correlated. Data in graphs G**–**I are represented as points and analysed using Spearman correlation (r) and its associated probability; **(G)** *Mecom* ^*Jbo/+*^ *n* = 26 XY pairs **(H)** *Fbxo11* ^*Jf/+*^ *n* = 43 XY pairs and **(I)** *Eda* ^*Ta*^ *n* = 48 XY pairs. Note zero counts WBCs were attributed a nominal value of one for graphical purposes the on log_10_ scale.

Recent work documents the cell response pathways involved in OM associated bulla mucosal hyperplasia; these include PI3K/AKT/PTEN (Lee et al., 2020) and heparin-binding epidermal growth factor driven cellular proliferation (Sakamoto et al., 2022). Bulla mucosal thickening is a commonly used measure of inflammation severity and we found a correlation between mucosal thickness and lumen WBC density measured in tissue sections. Bulla mucosae of *Mecom* ^*Jbo/+*^, *Fbxo11* ^*Jf/+*^, and *Eda* ^*Ta*^ are significantly thicker than wild-type controls (Figures 3A–C) and bulla cavity WBCs are elevated (Figures 3D–F). The reference ranges for healthy unaffected wild-type mucosa thickness were 12–28 μm, and 0–12 bulla cavity WBCs (5%–95% percentile range for *n* = 73 P21-P119 *Mecom* ^+/+^, *Fbxo11* ^+/+^ and FVB mice). We observed that 1/14 P22 *Mecom* ^*Jbo/+*^ bullae; 1/35 P57-P223 *Fbxo11* ^*Jf/+*^ bullae; 5/23 P21 and 5/25 P79-90 *Eda* ^*Ta*^ bullae were within normal limits for both parameters. In individual bullae, WBCs and mucosal thickness are positively correlated (with and without the inclusion of data from “normal bullae”) (Figures 3G–I). Within individual mice there is no correlation for these parameters between contralateral bullae (Table 1). Furthermore severity of bulla inflammation is highly variable and not significantly higher in older mice (>P57) compared with weaning aged (P21) mice (Figures 3A–F).

**TABLE 1:** Correlation between mucosal thickness and bulla cavity WBCs in contralateral bullae.

	Spearman r	95% confidence interval	*p* Value	Number of XY pairs
P21-P90 *Eda Ta* mucosa^a^	0.3907	−0.09160 to 0.7245	0.0982	19
P21-P90 *Eda Ta* WBC^a^	0.3272	−0.1633 to 0.6880	0.1715	19
P22-P84 *Mecom Jbo*/+ mucosa	0.1210	−0.4750 to 0.6409	0.6921	13
P22-P84 *Mecom Jbo*/+ WBC	0.3352	−0.2817 to 0.7560	0.2633	13
P21-P223 *Fbxo11 Jf*/+ mucosa	0.2198	−0.2891 to 0.6318	0.3808	18
P21-P223 *Fbxo11 Jf*/+ WBC	−0.1900	−0.6128 to 0.3174	0.4502	18

a After removal of bulla pairs which are within normal limits for mucosal thickness and WBC

### Epithelial and mucosal changes in chronic otitis media

**FIGURE 4:**
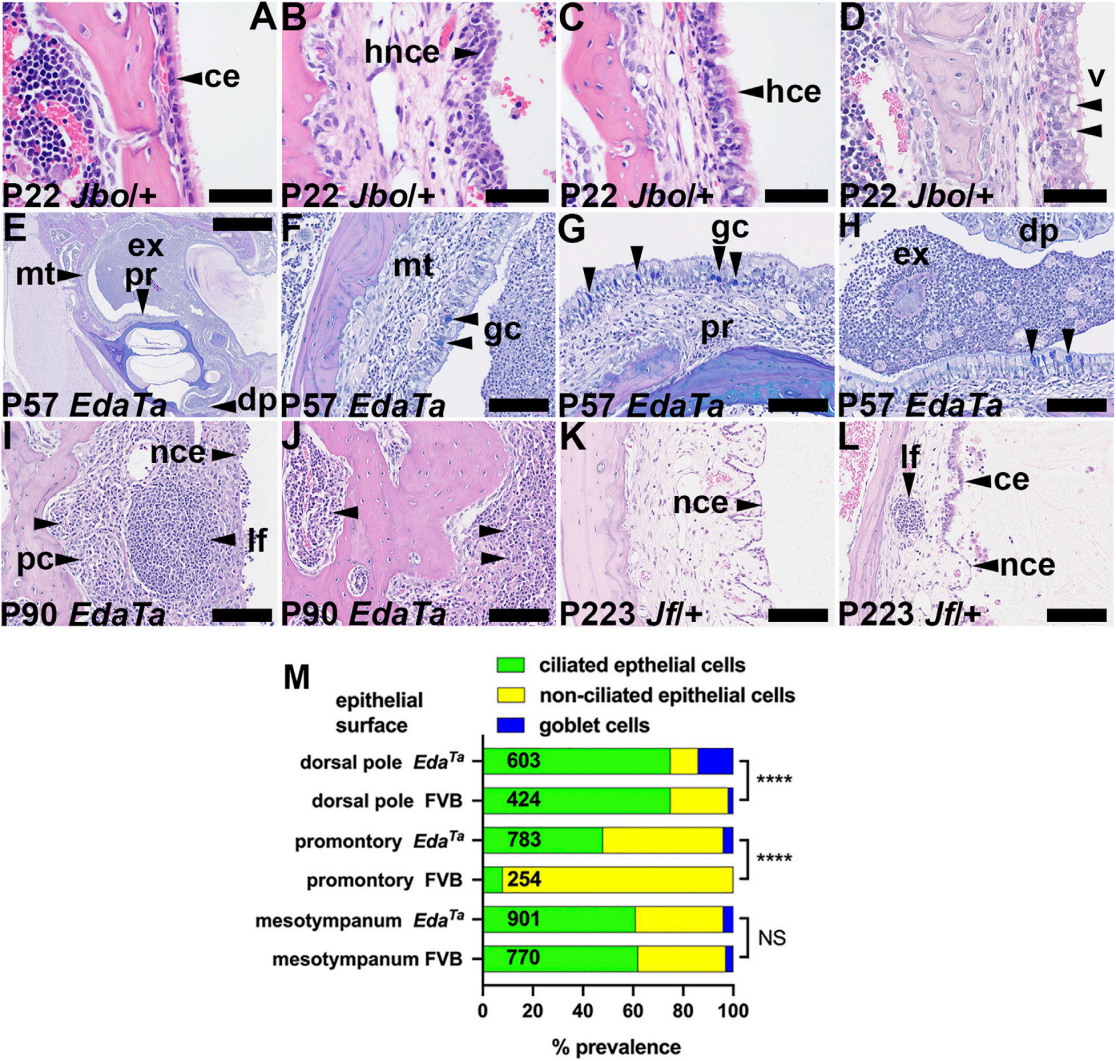
Epithelial and mucosal changes in otitis media. **(A–D)** Epithelial changes in P22 *Mecom* ^*Jbo/+*^ bulla mucosa. **(A)** Normal single layer of ciliated epithelium in a healthy ear, **(B)** hyperplastic non-ciliated epithelium **(C)** hyperplastic ciliated epithelium, **(D)** epithelial vacuolar degeneration. **(E–H)** AB-PAS stained sections of an *Eda* ^*Ta*^ bulla; **(E)** low power view of the bulla, **(F–H)** higher power images of selected sites shows goblet cells in the **(F)** mesotympanum, **(G)** promontory and **(H)** dorsal pole (arrowheads). **(I,J)** Mucosal and bone marrow plasmacytosis in a P90 *Eda* ^*Ta*^ bulla. **(K,L)** Micropolyps and lymphoid follicle in P223 *Fbxo11* ^*Jf/+*^ bullae. **(L)** Interface between ciliated and non-ciliated epithelium. Scale Bars: **(E)** 1,000 μm; **(F–L)** 100 μm; **(A–D)** 50 µm.ce, ciliated epithelium; dp, dorsal pole; ex, exudate; gc, goblet cell; hce, hyperplastic ciliated epithelium; hnce, hyperplastic non-ciliated epithelium; lf, lymphoid follicle; mt, mesotympanum; pc, plasma cells; pr, promontory; v, vacuole.**(M)** Epithelial population changes in inflamed *Eda* ^*Ta*^ bullae compared to normal FVB bullae assessed by the prevalence of ciliated, non-ciliated and goblet cells. Ciliated cells predominate in the dorsal pole region and the inflamed *Eda* ^*Ta*^ bulla has a significantly greater prevalence of goblet cells than the healthy FVB bullae. The promontory is chiefly covered by non-ciliated cells in FVB bullae, but there is an increase in ciliated cells and goblet cells in the inflamed *Eda* ^*Ta*^ bullae. The prevalence of non-ciliated, ciliated and goblet cells in the mesotympanum was comparable between *Eda* ^*Ta*^ and FVB bullae. Cell counts were made in a pairs of P57 *Eda* ^*Ta*^ and P79 FVB bullae. The numbers in the histogram bar are the total numbers of epithelial cells counted. Chi-square Test; NS, *p* > 0.05, ****p < 0.0001.

The bulla attic and promontory are lined chiefly with simple epithelium but there is a ciliated epithelium population in dorsal pole adjacent to the oval window. The hypotympanum comprises a pseudostratified ciliated epithelium with goblet cells and areas of non-ciliated cells (Thompson and Tucker, 2013; Luo et al., 2017; Tucker et al., 2018). Bulla epithelial populations alter in response to inflammation (Tucker et al., 2018; del-Pozo et al., 2109a) and we performed a histological analysis to measure these changes. The mesotympanum epithelium comprises non-ciliated simple epithelium as well as pseudostratified ciliated epithelium (Figure 4A) with goblet cells. Inflammatory changes include hyperplasia of the non-ciliated and ciliated cells (Figures 4B,C). Ciliated cells can be hypertrophic columnar cells and undergo vacuolar degeneration (Figure 4D). The promontory in P57 *Eda* ^*Ta*^ bullae shows ciliated cell hyperplasia and a small population of goblet cells (Figures 4E–G; Supplementary Table S1). Goblet cells are also increased dorsal pole region (Figure 4H; Supplementary Table S1). Away from the auditory tube ostia the goblet cell hyperplasia was modest (Figure 4M; Supplementary Table S1). Normal healthy C57BL/6J bulla mucosa has transcriptomic signatures of T-cell, NK cell and B cells (Ryan et al., 2020). In mice, nasal associated lymphoid tissue is the major inductive site of antigenic responses to nasopharyngeal bacteria but the chronically inflamed bulla develops a resident population of lymphocytes (Sabirov and Metzger, 2008a; Sabirov and Metzger, 2008b). Chronically inflamed bulla mucosa can have scattered lymphoid follicles (Figures 4I,L), micropolyps (Figure 4K) and one P90 *Eda* ^*Ta*^ mouse had bilateral mucosal and bone marrow plasmacytosis (Figures 4I,J).

### Tympanic temporal bone remodelling and sclerosis

**FIGURE 5:**
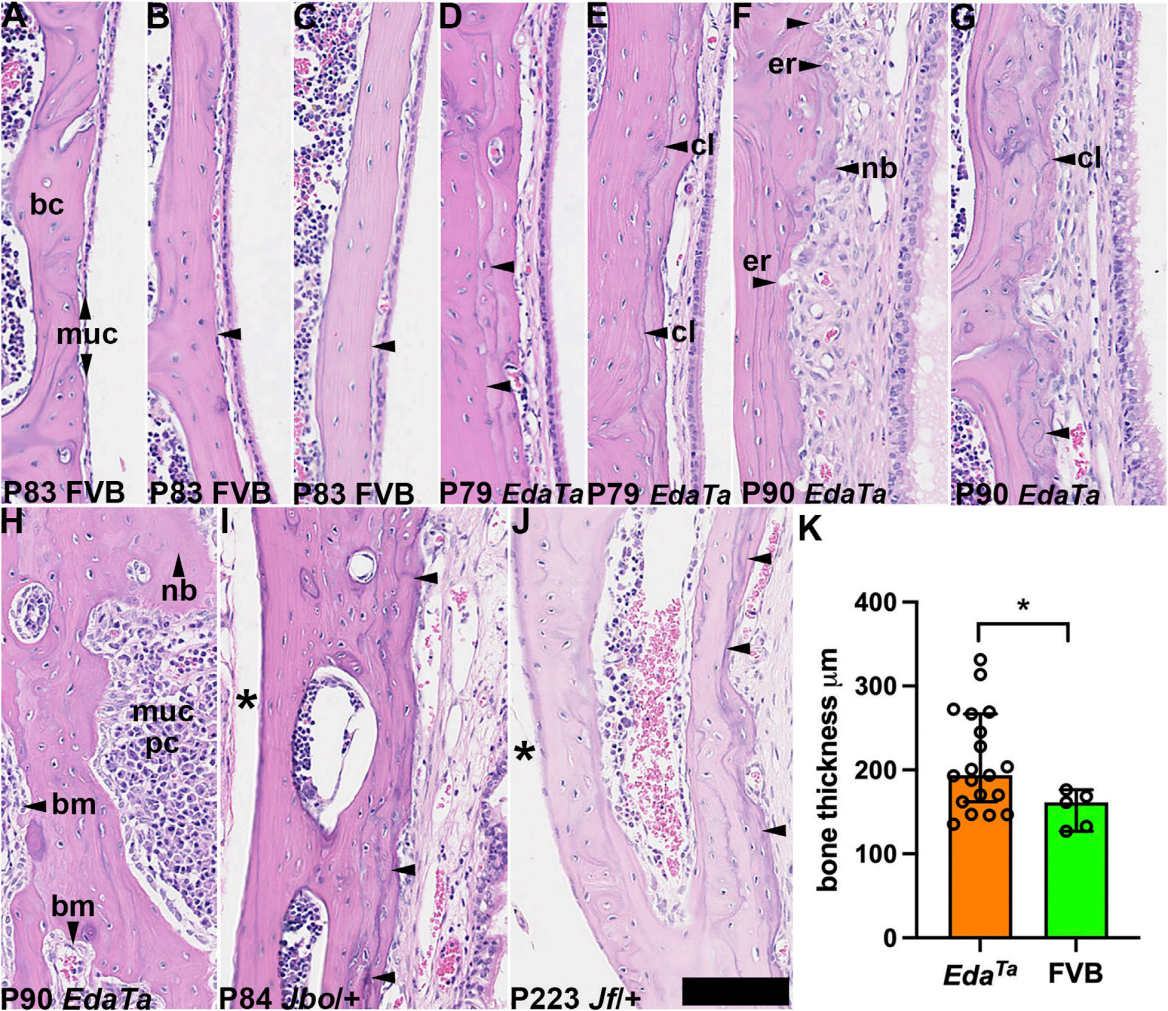
Tympanic temporal bone changes associated with mucosal inflammation. Dorsal plane sections of tympanic temporal bulla bone cortices, with mucosa on the right. **(A–C)** Examples of P83 FVB temporal bone with a slender mucosa and relatively smooth bone cortex margin (arrowheads). **(D–H)** P79-P90 *Eda* ^*Ta*^ mice. **(D,E)** The bone cortex margin is smooth in a minority of *Eda* ^*Ta*^ mice which have minimal changes in the overlying mucosa, note cement lines (arrowheads). **(F–H)** Examples of other *Eda* ^*Ta*^ bullae where the mucosa is thickened with variable inflammatory cell infiltration, angiogenesis and epithelial hyperplasia. **(F)** Focal micro-erosion of the cortex margin and deposition of new bone; **(G)** cement lines are wavy. **(H)** Diffuse plasma cell infiltration of the mucosa, the bone is focally thickened (vertical arrowhead). **(I)** P84 *Mecom* ^*Jbo/+*^ and **(J)** P223 *Fbxo11* ^*Jf/+*^ tympanic temporal bones show increased density of cement lines on the mucosal cortex compared with the medial cortex (asterisk). **(K)** *Eda* ^*Ta*^ tympanic temporal bone is significantly thickened compared to FVB mice. Data in graph K are represented as data points, the histogram bar is the median and the error bars 95% confidence intervals; *n* = 5 P81-P84 FVB and *n* = 19 P79-P90 *Eda* ^*Ta*^ temporal bones and analysed using Mann Whitney test: *p < 0.05. Thirteen of 19 *Eda* ^*Ta*^ temporal bones had histological evidence of bone erosion/deposition but this was not observed in FVB temporal bones (0/5) (*p* = 0.0109, Fisher’s exact test). bc, bone cortex; bm, bone marrow; cl, cement line; er, erosion; muc, mucosa; nb, new bone. Scale Bar **(J)** 50 μm; all the images shown at the same magnification.

Bone sclerosis (Berry et al., 1994) and neo-ossification (Noben-Trauth and Latoche, 2011) have been reported in other mouse models of otitis media. In contrast to healthy P83 FVB bullae (Figures 5A–C), P79-90 *Eda* ^*Ta*^ bullae with thickened inflamed mucosae have wavy cement lines and focal micro-erosions in the mucosal aspect of the temporal bone cortex that are indicative of bone regression and new bone deposition (Figures 5D–H). P84 *Mecom* ^*Jbo/+*^ and P223 *Fbxo11* ^*Jf/+*^ mice also showed wavy cement lines (but no micro-erosions) on the mucosal cortex but not on the medial cortex (Figures 5I,J). Tympanic bone thickness in P79-P90 *Eda* ^*Ta*^ was significantly greater than that in P81-P83 FVB (Figure 5K). These bone changes appear age-related and were not observed in weaning aged *Mecom* ^*Jbo/+*^ and *Fbxo11* ^*Jf/+*^ mice or in P57-P119 *Fbxo11* ^*Jf/+*^ mice.

## Discussion

We report that mice have trans-cortical vessels in the tympanic temporal bone that connect the marrow to the middle ear mucosa lining the bulla cavity. *Mecom* ^*Jbo/+*^ and *Fbxo11* ^*Jf/+*^ mice with chronic otitis media have suppurative and serous bulla fluids respectively but neither have substantial changes in their peripheral blood leukogram compared to wild-type littermates. This suggests a systemic immunological response does not occur to meet neutrophil demand. In this work we examine evidence to support TCVs having a role in the pathogenesis of otitis media through delivery of myeloid cells to the mucosal lining and air filled bulla cavity.

### Temporal bone channels and trans-cortical vessels

The human temporal bone has a squamous tympanic, a petrous, and a mastoid part; the mastoid has a large number of air cells (Isaacson, 2018) whereas the mouse tympanic bone lacks a mastoid (Carretero et al., 2017). Embryonic mesenchyme that fills the foetal bulla cavity regresses to form the adult air-filled bulla. In normal human temporal bones, mesenchyme occupies 20% of the bulla cavity at birth and largely regresses by 12 months of age (Takahara and Sando 1987) and bulla cavitation defects can be associated with OM (Jaisinghani et al., 1999). Bone channels in foetal and neonatal human temporal bone connect marrow with bulla mesenchyme allowing WBCs to enter the mesenchyme but no specific vessels have been described (Linthicum and Tian, 1997). The prevalence of marrow-mesenchyme connections is a risk factor for bacterial infection from the ear causing meningitis (Terao et al., 2011). The human mastoid bone has micro-channels containing venules and arterioles that appear to have periosteal origin and provide a blood supply to the mucosa. This network of micro-channels in compact and trabecular bone connect to mastoid air cells and may facilitate gas exchange and middle ear volume regulation (Cros et al., 2013, 2015). However it has not been established whether these TCVs connect with the marrow.

In the mouse normal bulla mesenchyme regression occurs at ∼ P11 (Del-Pozo et al., 2019a) before temporal bone TCVs appear at ∼ P13 and TCVs persist in adults. The oldest mice we examined (P223) had bone marrow but full age range over which this is present remains to be determined. There is extra medullary haematopoiesis in the mucosa of P21 mice with OM and this may represent a response to bulla inflammation and/or hypoxia (Yang et al., 2020).

### Bulla cavity myeloid cell populations and renewal

Signalling molecules from the inflamed brain may preferentially reach the skull marrow and facilitate myeloid cell mobilization (Herisson et al., 2018). Signalling molecules from the inflamed bulla are likely to initiate the observed changes in epithelial populations and bone remodelling, and by analogy myeloid cell mobilization from temporal bone marrow. Under resting conditions >98% of murine neutrophils are located bone marrow and these act as a reservoir to respond to acute stress such as infection (Day and Link, 2012). The marrow sinusoidal endothelium acts as a facilitator/regulator of neutrophil release from the bone marrow (Hassanshahi et al., 2019). CXCL12 (=SDF)/CXCR4 binding is a neutrophil retention signal (Day and Link, 2012) and CXCL12 declines in skull marrow after stroke (Herisson et al., 2018). Other signalling molecules involved in myeloid cell chemoattraction, activation and release are detected in otitis media. CXCL1 (KC) and CXCL2 (GRObeta/MIP2) chemoattract and activate neutrophils and CXCL2 binding to CXCR2, directs neutrophil release (Day and Link, 2012). CCL2 and CCL7 regulate B-lymphocyte and monocyte mobilization (Hassanshahi et al., 2019). Factors regulating haematopoiesis include IL-17A acting with TNFa to promote secretion of G-CSF (CSF3) (Bosteen et al., 2014) and metalloproteinases and their inhibitors alter the bioavailability of niche factors such as CXCL12, SCF (Kit Ligand; KITLG), TGFb, and VEGF (Saw et al., 2019). Expression of these genes and protein products as well as haematopoietic factors M-CSF (CSF1) and GM-CSF (CSF2) are detected in human COME fluids (Smirnova et al., 2005; Juhn et al., 2008; Sekiyama et al., 2011; Val et al., 2018; Bhutta et al., 2020) and AOM induced in wild-type inbred mouse strains with *Haemophilus influenzae* (MacArthur et al., 2011,^2013^; Preciado et al., 2013; Hernandez et al., 2015; Trune et al., 2015; Granland et al., 2020). In this challenge model, AOM is self-limiting. *Mecom* ^*Jbo/+*^ and *Fbxo11* ^*Jf/*+^ develop chronic OM spontaneously and WBCs from the bulla cavity show upregulated gene expression of CCL2, CCL7, CXCL2, TNFa, and VEGF (ranging from 50–3,000 fold) compared peripheral blood WBCs. CXCL1 and CXCR4 (ranging from 5–70 fold) are also upregulated in *Mecom* ^*Jbo/+*^ (Cheeseman et al., 2011). However, relative gene expression levels in bone marrow and the inflammatory lesion remain to be determined.

*Mecom* ^*Jbo/+*^ bulla exudates are >10-fold more cellular than *Fbxo11* ^*Jf/+*^ effusions. Neutrophils predominate in *Mecom* ^*Jbo/+*^ suppurative exudates but macrophages, monocytes and lymphocytes predominate in *Fbxo11* ^*Jf/+*^ serous effusions (Cheeseman et al., 2011; Del-Pozo et al., 2019a). In this study we found comparable results: ∼10-fold greater WBC numbers in *Mecom* ^*Jbo/+*^ than *Fbxo11* ^*Jf/+*^ bulla fluids and FACS analysis of live CD45^+^ gated cells showed the proportions of neutrophil and monocyte lineage cells (monocytes and macrophages) is 5% and 22% in *Fbxo11* ^*Jf/+*^ and 90% and 4% in *Mecom* ^*Jbo/+*^ mice. We note that the large lipid-laden foamy macrophages seen in histology sections (Cheeseman et al., 2011; Azar et al., 2016) cannot be cell sorted, probably because of their fragility and are under-represented in FACS analysis. Macrophages remove senescent neutrophils from the circulation and sites of tissue inflammation; this is efficiently achieved in the spleen, liver and bone marrow (Rankin, 2010). However re-uptake into the circulation from a hollow cavity rather than vascularised tissue seems less likely.

The lifespan of mouse neutrophils is 0.75 days in the circulation (Pillay et al., 2010) and their survival is prolonged in inflammatory hypoxic environments through apoptosis inhibition and maintenance of ATP generation (Nathan and Ding, 2010). Nevertheless the hypoxic population of *Mecom* ^*Jbo/+*^ neutrophils in bulla fluids has a lower percentage of viable cells (12%–22%) and higher apoptotic cells (63%–79%) than the normoxic population (63%–79% viable and 11%–45% apoptotic); and 7% of neutrophils are necrotic (Cheeseman et al., 2011). Apoptotic neutrophils that are not effectively cleared by macrophages will undergo necrosis in the bulla cavity. The products of host cell necrosis and bacterial re-infection act as inflammatory stimuli which produce a state of non-resolving inflammation (Nathan and Ding, 2010).

We found that the systemic depletion of macrophages and monocytes in *Mecom* ^*Jbo/+*^ mice over 6–8 days with clodronate liposomes did not alter the bulla macrophage and monocyte, and neutrophil populations expressed as a percentages of CD45^+^ cells. We infer chlodronate liposomes do not penetrate the bulla epithelial barrier and that resident bulla macrophages and monocytes escape toxicity and are relatively long-lived. Neutrophil numbers in the bulla cavity will be a balance between recruitment, lifespan of cells, apoptosis and macrophage clearance, cell necrosis, and clearance *via* the auditory tube. There is ciliated cell hyperplasia in the inflamed *Mecom* ^*Jbo/+*^ bulla (Del-Pozo et al., 2019a; and in this study) but a morphometric analysis of the chronically inflamed *Eda* ^*Ta*^ bulla, which also has a suppurative exudate, revealed the degree of ciliated cell and goblet cell hyperplasia is modest. This represents a repair response to injury caused by infection/inflammation (Tucker et al., 2018) but is unlikely to markedly improve mucociliary clearance as drainage through the narrow auditory tube is restricted. The proportion of neutrophils that are necrotic is particularly high when *Mecom* ^*Jbo/+*^ bulla fluids have acute bacterial infection such as in experimental NTHi challenge and few macrophages contain intra-lysosomal apoptotic bodies (Hood et al., 2016). Host cell (neutrophil) necrosis provides a stimulus for neutrophilic exudation in *Mecom* ^*Jbo/+*^ bulla fluids but pyogenic bacterial infection may also play a part and we cultured opportunistic pathogens including *Proteus mirabilis*, *Staphylococcus* sp. and *E. coli* which are common nasopharyngeal commensals in our colony mice (Azar et al., 2016; Del-Pozo et al., 2019a). We found the total bacterial load was positively correlated with WBC numbers. The degree of inflammation varies between contralateral bullae and between mice of different ages. We infer inflammation may wax and wane and may be re-initiated by a new wave of bacterial infection.

A wider panel of FACS markers has been used to characterise myeloid and lymphoid cell subsets in *Fbxo11* ^*Jf/+*^ bulla fluids into neutrophils, dendritic cells (progenitor DC and mature DCs) macrophages and monocytes and lymphocyte populations including T helper, T regulatory, B cells and NK cells (Vikhe et al., 2020).

### Limited changes in peripheral blood leukograms

*Mecom* ^*Jbo/+*^ mice have highly cellular neutrophil (90%) rich bulla exudates whereas *Fbxo11* ^*Jf/+*^ have low cellularity effusions (5% neutrophils) and we examined evidence that differing demand for neutrophils is associated with peripheral blood leukogram change. *Fbxo11* ^*Jf/+*^ and *Mecom* ^*Jbo/+*^ mice have normal WBC counts compared to wild-type controls (International Mouse Phenotype Consortium https://www.mousephenotype.org/) and *Mecom* ^*Jbo/+*^ mice do not exhibit neutrophilia or differences in immature and mature neutrophils compared with wild-type littermates (Parkinson et al., 2006). In the current study we found no statistical differences in the leukograms and only minimal changes for spleen WBC differentials for *Fbxo11* ^*Jf/+*^ and *Mecom* ^*Jbo/+*^ mice compared to their respective wild-type littermates. In contrast, other studies report elevated blood neutrophils in *Fbxo11* ^*Jf/+*^ at P43-P44 (but not at P121-P127) (Hardisty et al., 2003) and elevated blood neutrophil, macrophage, DC CD811 type, and NK cell differentials at 8 weeks (Vikhe et al., 2020). The margin of neutrophil increase (*Fbxo11* ^*Jf/+*^ 19.3% versus 16.6% wild-type littermates) in the latter study was smaller than the difference between males and females in inbred mouse strains (Peters et al., 2002) and the percentage of neutrophils in *Fbxo11* ^*Jf/+*^ bulla fluid (10.3%) is actually lower than blood. Other elevated blood myeloid classes represent small WBC populations; macrophages (<0.6%) and DC CD811 type (<3.3%) (Vikhe et al., 2020).

In summary leukogram changes can occur in children with AOM and acute infection but often AOM co-exists with other acute inflammatory conditions; leukogram change is less apparent in chronic OM. Leukocytosis is absent in *Fbxo11* ^*Jf/+*^ and *Mecom* ^*Jbo/+*^ mice with chronic OM (International Mouse Phenotype Consortium) and data for WBC differential counts is not entirely consistent. The lack of detailed knowledge about the lifespan and clearance of neutrophils in the bulla cavity makes it difficult to assess the true demand for myeloid cell recruitment but it is likely much higher for *Mecom* ^*Jbo/+*^ mice with suppurative exudates. The serous *Fbxo11* ^*Jf/+*^ bulla fluids may arguably arise through transudation and trapping of fluid caused by bulla adhesions (Del-Pozo et al., 2019a). *Mecom* ^*Jbo/+*^ mice have dysregulated NF-kB-dependent inflammatory responses and higher neutrophil infiltration in NTHi lung challenge (Xu et al., 2012) and we found colonization of bulla fluid by endemic nasopharyngeal bacteria is associated with increased neutrophil numbers. We did not detect overt leukogram change in either mouse strain but TCVs in temporal bone could provide a mechanism for preferential mobilization of myeloid cells and mitigate a systemic immunological response. When leukogram change does occur in acute severe OM it may be the result of myeloid cells mobilized from temporal bone entering the general circulation rather than, or in addition to, mobilization from distant sites. Further experiments using cell tracing are necessary to resolve these questions.

### Inflammation and temporal bone remodelling

Otitis media causes sclerosis of the human temporal bone (Schrimpf et al., 1982; Lee et al., 2008; Juliano, 2018) and we found *Mecom* ^*Jbo/+*^, *Fbxo11* ^*Jf/+*^, and *Eda* ^*Ta*^ mice show temporal bone remodelling which is a feature in other mutant mice with OM (Berry et al., 1994; Noben-Trauth and Latoche, 2011). RANKL/RANK signalling is involved in coupling bone resorption and formation (Takegahara et al., 2022) and this pathway is upregulated in bulla cavity WBCs in human COME (Bhutta et al., 2020). Experimental arthritis increases the density of TCVs in mouse tibial bone (Grüneboom et al., 2019), but our simple histological survey was not as sensitive as transmission electron microscopy to detect this change. It is possible that progressive bone sclerosis in chronic otitis media eventually causes a decline in TCV density.

### Bone marrow activity, trans-cortical vessels and longitudinal patterns of otitis media presentation

The haematopoietic activity of the human temporal marrow has not been studied in the context of otitis media but MRI imaging shows mastoid bone marrow disappears by 1 year (Sano et al., 2007) and the adult petrous apex marrow has a fatty signal (Chapman et al., 2011). The cellularity of bone marrow in general changes with age. Infants 1–12 months-old have 80%–90% bone marrow cellularity; children 2–5 years, 60%–80%; 6–12 years, 50%–70%, and 12 years to adults, 40%–60%. The myeloid to erythroid ratio is 5–12: 1 in infants and, from 2 years of age onwards, the M:E ratio is 3–4 : 1 (Proytcheva, 2013). The peak occurrence of AOM is between 6 months and 2 years (Danishyar and Ashurst, 2020) and COME peaks at 2 years then declines progressively to 12 years (Suarez Nieto et al., 1983). This raises the question whether the pattern and presentation of otitis media is related to changes in bone marrow function and TCVs. Evidence for one possible link comes from the historic use of low-dose X-ray irradiation to treat cervical adenitis (=lymphadenitis), mastoiditis and chronic OM (see review by Calabrese and Dhawan, 2014). The mechanistic basis of X-ray treatment is to establish an anti-inflammatory phenotype characterised by decreased nitric oxide/inducible nitric oxide synthase, reactive oxygen species, TNF-a and adhesion of WBCs to endothelial cells; increases in heme oxygenase and TNF-b; and activation of the transcription factors NF-kB and AP-1 (Calabrese and Dhawan, 2014). Irradiation of bone also suppresses haematopoiesis (Green and Rubin, 2014) and produces a significant reduction in TCVs (Grüneboom et al., 2019). Taken together X-ray irradiation could limit myeloid cell mobilization contributing to the therapeutic response.

The difference in size of the bulla in mice and humans may impact on the presentation of otitis media. The mouse middle ear attains its full adult size (∼5.5 µl) by 3 weeks-of-age (Huangfu and Saunders 1983) and will have a relatively high temporal bone area surface to volume ratio for TCVs to deliver myeloid cells. In mouse mutant strains that develop chronic otitis media spontaneously, the failure to clear WBCs and inflammatory stimuli may create a pro-inflammatory feedback loop that is responsible for delayed resolution seen in these strains (Bhutta et al., 2017). The size of the human middle ear is established at birth and is 0.5–1.0 ml; the mastoid is considerably larger (10.3 ml) (Dirckx et al., 2013) but its bone marrow disappears by 1 year (Sano et al., 2007). The lower temporal bone area to volume ratio may limit TCV density. If the natural decline bone marrow myeloid cell content with age results in lower mobilisation of myeloid cells, this may conceivably contribute to the decline in incidence of human COME from 2 to 12 years of age and its spontaneous resolution.

The standard surgical treatment for COME is to drain the bulla effusion and insert a ventilation tube in the tympanic membrane to re-aerate the bulla cavity. Surgical incision of the tympanic membrane in *Mecom* ^*Jbo/+*^ mice and removal of fluid reduces recurrence of effusion, mucosal thickening and tissue hypoxia (Bhutta et al., 2014). It may be that removal of bulla inflammatory fluids acts in part by halting the cycle of recruitment of marrow myeloid cells *via* TCVs.

## Conclusion

The mouse bulla temporal bone, like other skull flat bones, has trans-cortical vessels and these connect marrow and bulla mucosal vasculature. We hypothesize the preferential recruitment of myeloid cells into the inflamed bulla from temporal bulla bone marrow may mitigate the need for a systemic immunological reaction and limit changes observed in the peripheral leukogram. Further studies are needed to define the density of temporal bone TCVs in mice using techniques such as transmission electron microscopy and tracer studies, and to evaluate the functional contribution of temporal bone marrow to bulla mucosal circulation. Further study of the mechanisms of myeloid cell mobilization from the temporal bone marrow in mice and bulla inflammatory stimuli which promote recruitment may reveal important targets for pharmacological intervention.

## Data Availability

The original contributions presented in the study are included in the article/Supplementary Material, further inquiries can be directed to the corresponding author.
