# Supplementary figures and images for: Trans-cortical vessels in the mouse temporal bulla bone are a means to recruit myeloid cells in chronic otitis media and limit peripheral leukogram changes
Source: Front Genet. 2022 Sep 28;13:985214. doi: 10.3389/fgene.2022.985214 (PMC9555619; doi:10.3389/fgene.2022.985214)

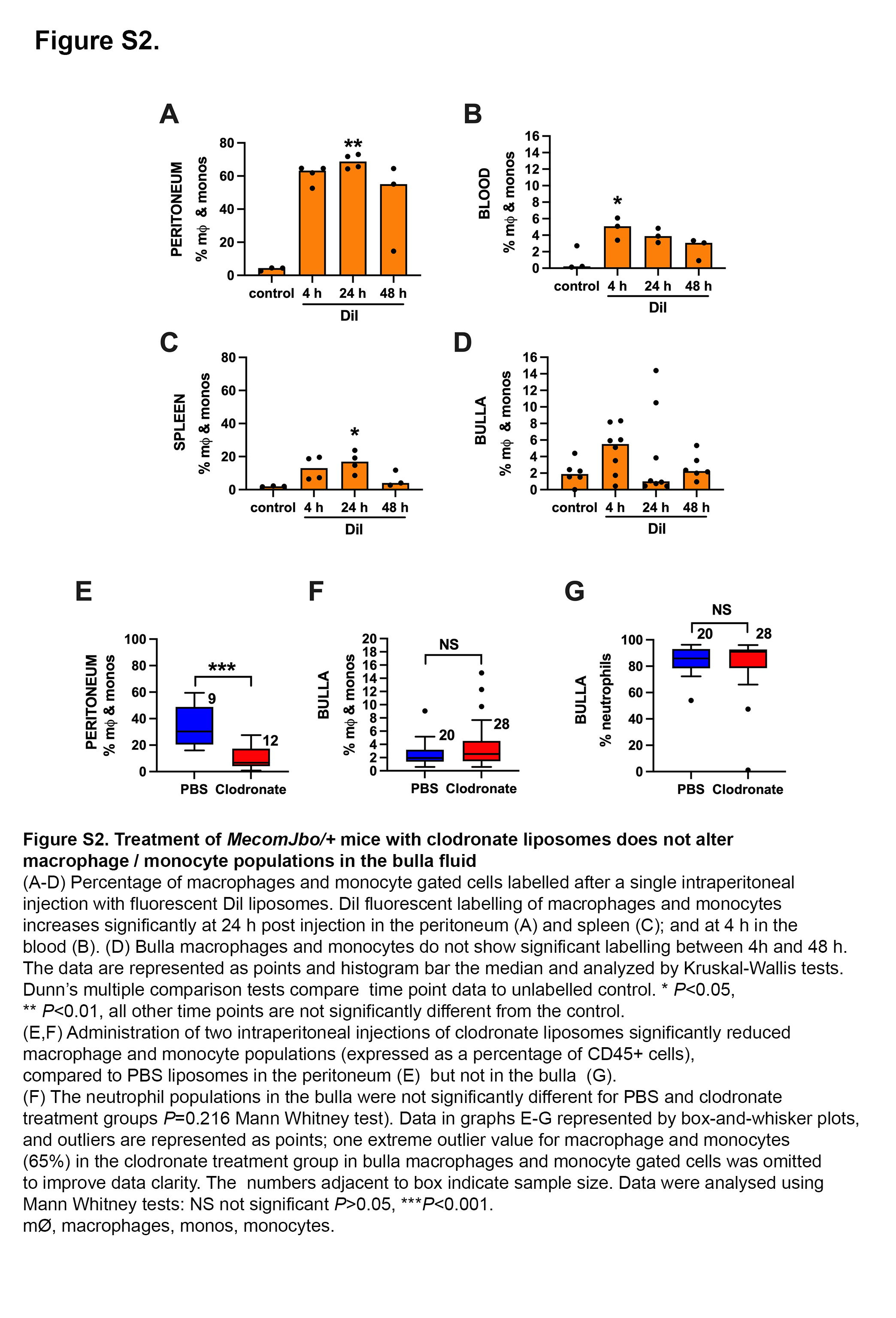

Supplement: Supplementary file 1 [file Image2.TIF]

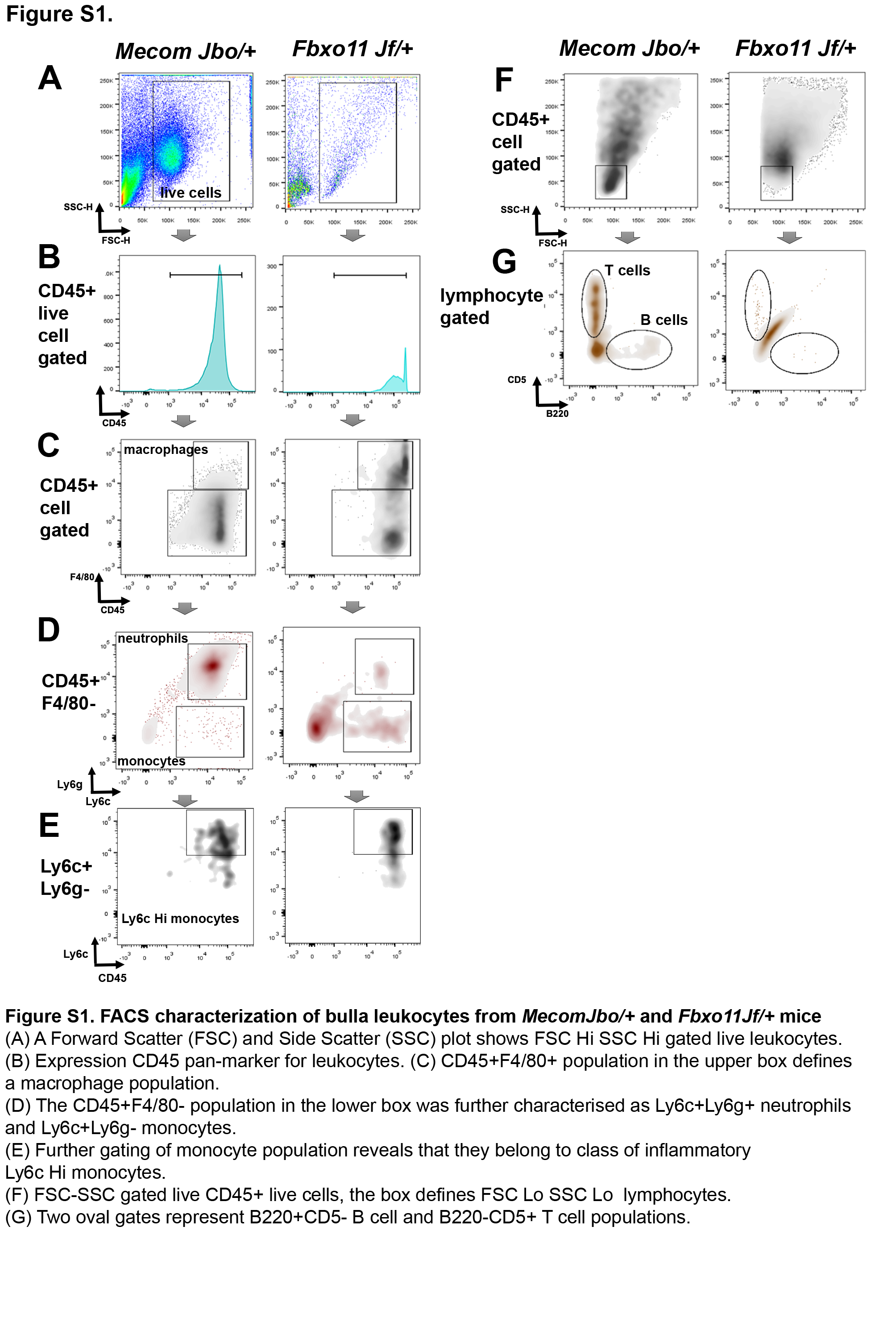

Supplement: Supplementary file 2 [file Image1.TIF]
